# Determinants of self-rated health in women: a population-based study in Armavir Marz, Armenia, 2001 & 2004

**DOI:** 10.1186/1475-9276-7-25

**Published:** 2008-12-12

**Authors:** Anahit Demirchyan, Michael E Thompson

**Affiliations:** 1 Center for Health Services Research and Development, American University of Armenia, Yerevan 0019, Armenia; 2 Department of Public Health Sciences, University of North Carolina at Charlotte, NC, USA

## Abstract

**Background:**

The former soviet Republic of Armenia entered a turbulent and long-lasting economic transition when it declared its independence in 1991. This analysis sought to identify the determinants of poor self-rated health as an indirect measure of health status and mortality prognosis in an adult female population during a period of socio-economic transition in Armenia.

**Methods:**

Differences in self-rated health in women respondents were analyzed along three main dimensions: social, behavioral/attitudinal, and psychological. The data used were generated from cross-sectional household health surveys conducted in Armavir *marz *in 2001 and 2004. The surveys utilized the same instruments and study design (probability proportional to size, multistage cluster sampling with a combination of interviewer-administered and self-administered surveys) and generated two independent samples of households representative of Armavir *marz*. Binary logistic regression models with self-rated health as the outcome were fitted to the 2001 and 2004 datasets and a combined 2001/2004 dataset.

**Results:**

Overall, 2 038 women aged 18 and over participated in the two surveys (1 019 in each). The rate of perceived "poor" health was relatively high in both surveys: 38.1% in 2001 and 27.0% in 2004. The sets of independent predictors of poor self-rated health were similar in all three models and included severe and moderate material deprivation, probable and possible depression, low level of education, and having ever smoked. These predictors mediated the effect of women's economic activity (including unemployment), ethnicity, low access to/utilization of healthcare services, and living alone on self-rated health.

**Conclusion:**

Material deprivation was the most influential predictor of self-rated health. Thus, social reforms to decrease the gap between the rich and poor are recommended as a powerful tool for reducing health inequalities and improving the health status of the population.

## Background

The Republic of Armenia is a small, landlocked, Transcaucasian country with an estimated 3.2 million population [1] situated along the northeastern Armenian Highlands. Over the last two decades, Armenia has experienced many dramatic changes that negatively impacted the health of its population. Major cataclysms included the disastrous earthquake in 1988 with an estimated 25 000 deaths, 130 000 injured persons, and over 500 000 homeless people; the Karabagh conflict in early 1990s, which resulted in an estimated 15 000 deaths and 310 000 refugees and displaced persons in Armenia and coincided with a long-lasting economic blockade and ensuing energy crisis; the difficult transition from the Soviet system to a market economy; and, in response to these challenges, considerable emigration, especially among the labor force [2]. Annual surveys conducted since 1996 revealed that over half of the population were impoverished until the early 2000s: in 2001, 34.9% of the population were poor and 16.0% very poor (monthly per-capita expenditures below the actual minimal food basket). In 2004, these proportions decreased to 34.6% and 6.4% respectively [3,4].

In the mid 1980s, life expectancy in Armenia was the highest among the Soviet republics (73.3 years in 1986, 70.5 for males and 75.7 for females). By 1991, the early years of independence, it had fallen to 70.4 years (67.4 for males and 73.3 for females), only returning to its previous level in 2004 (73.4 years) [4]. As infant mortality rates improved slightly during this period, the observed decrease in life expectancy was due to increased rates of cardiovascular disease, cancer, diabetes, and tuberculosis [4] coupled with drastic decreases in the utilization of health care services. These changes resulted from an inability to pay for services [5] in a health care system that was now effectively based on out-of-pocket payments. These informal payments were estimated to constitute about 80% of all system resources [6]. Despite these changes, life expectancy remained appreciably higher than the average for the CIS region (67 years) [7].

Significant changes also occurred in Armenia's demographic profile. The population "aged" rapidly due to the combined effects of emigration and a precipitous decline in the birthrate to a net reproductive rate of 1.7 (below the threshold of 2.1 needed to maintain a population) [8]. Meanwhile, the observed crude mortality rate had increased by 40% during the past two decades [4,9]. Similar trends were observed in many other Eastern European and former Soviet Republics during this protracted period of socio-economic transition [10-14] and, thus, may have common underlying mechanisms.

There are no published studies investigating the main determinants of the changes in population health in Armenia, but increasing evidence from other former Soviet Republics and countries of Central and Eastern Europe experiencing the same transition to market economy suggests a deterministic role of socioeconomic and psychosocial environments on changes in health status and mortality trends [10-16]. Although the timing of these cataclysmic events and the ensuing socio-economic transition coincided with the changes in population health status, no attempt has been made to study the strength of association between the two or to determine the population groups most vulnerable to these changes.

### Study Aims

This analysis sought to identify the determinants of health status in an adult female population during a period of socio-economic transition in Armenia. Self-rated health was taken as a proxy for health status and mortality prognosis, as it is a well-established and strong predictor of morbidity and physical functioning, as well as a reliable and valid independent predictor of mortality both for women and men [17-20]. Moreover, self-rated health is known to be a comprehensive measure reflecting not only the absence of disorders, but also the range of biological, psychological, and social components that constitute the well-being of an individual [10,12,13,21-25]. While several studies have shown that self-rated health inequalities are largely similar for men and women [26-28], the survey protocol focused on recruiting women as women were considered to be more informed about health and health care practices relating to children and to other members of the household and could therefore provide the richest information needed to address other study aims. Furthermore, focusing on women was guided by the recognition that far more attention has been directed at excess male mortality in transition countries and that further research focusing more specifically on women's health was recommended [14].

Differences in self-rated health were analyzed along three main dimensions: social, behavioral/attitudinal and psychological, since prior studies had shown that these dimensions provided a comprehensive set of determinants of self-rated health status [11-14,16,21,25,29]. The study also examined the effect of a health status correlate, a combined variable of physical health status measures, on self-rated health.

## Methods

### The Surveys

The Armavir Household Heath Survey was conducted in two successive stages in 2001 and 2004 by the Center for Health Services Research and Development (CHSR) of the American University of Armenia (AUA). These studies were prospectively designed to evaluate the impact of a US-Armenia community health partnership between the University of Texas Medical Branch (UTMB, Galveston) and the Armavir Health Department that was administered by the American International Health Alliance with funds provided by the United States Agency for International Development. The cross-sectional surveys utilized the same study design and instruments and generated two independent samples of households representative for Armavir *marz*, one of the eleven administrative subdivisions of Armenia with a population of 260 000 [1] and demographic/health indicators close to the average for Armenia [4,8]. The study reports [30,31] provide the only available and reliable data on these surveys.

### The Sample

The surveys utilized a probability proportional to size, multistage cluster sampling design. Each cluster consisted of 10 respondent households. Interviewers tracked the results of each household visit to document the number of eligible respondents contacted, the explicit refusal rate, and the reason(s) for non-response in securing the requisite sample. The sampling frame for the randomly selected cluster start points was comprised of lists of children aged 2–4 years available from local primary health care facilities. These lists were believed to be the most complete in terms of population coverage and more accurate than other available population listings [32]. The density of children was presumed to reflect the overall population density of the area. While all women at least 18 years of age living in the household were considered as eligible respondents, first priority went to women with a child under 10 years of age, second priority to married women, and third priority to other women. If more than one woman living in a household qualified in the same priority group, the respondent was randomly selected using a random number table provided to interviewers. The preference for respondents with young children was necessary to ensure better data related to child care and reproductive health objectives of the assessment. Comprehensive health status and health system utilization data were collected from each sampled household through a mix of interviewer-administered and self-administered questionnaires. SPSS 11.0 statistical software was used for double entry and subsequent cleaning of the data. The survey protocol was reviewed and approved by the IRBs of the AUA and UTMB. The main body of analyses presented in this paper is based on the combined sample of the two surveys consisted of 2 038 female respondents aged 18 and over. However, to detect the differences between 2001 and 2004 data, separate analyses of each dataset were also conducted.

### Respondents

A total of 1 019 households from 59 populated areas participated in the survey in April, 2001, and the same quantity from 63 populated areas in May, 2004. The refusal rate was 7.4% in 2001 and 12.5% in 2004. Incomplete surveys constituted 0.7% in 2001 and 3.2% in 2004. Additional file 1 presents the demographic profile of the 2001 and 2004 survey respondents. Overall, the samples were comparable to the population from which they were drawn with expected measures stable over the survey interval. The vast majority of respondents were Armenian, over two-thirds lived in rural areas, most had a secondary education or less, and few reported smoking or drinking regularly. The only exception was the mean age of respondents, which was similar at the two surveys (35.6 in 2001 and 36.0 in 2004), but somewhat lower than the mean age (40.6) of adult female population of Armavir marz [1]. This difference was due to the under-representation of older women in the samples inherent in the selection criteria described above that favored selecting a mother of a young child. Between 2001 and 2004, unemployment dropped and material deprivation decreased significantly. Utilization of/access to primary healthcare services increased as did interest in a healthy lifestyle. The prevalence of depression decreased and perceived health status improved. Exposure to violence decreased while distrust of the police and public safety concerns increased.

### Variables

The dependent variable, *self-rated health *was measured by a single question taken from the SF-36 questionnaire [33]: "How would you describe your health in the last month?", and then dichotomized as "poor" versus all other categories (e.g., "not poor."). A self-rated health status correlate variable named *physical health *was constructed on the basis of a cumulative score generated from the responses to the following items: presence of one or more self-reported chronic health conditions, magnitude of bodily pain experienced by the respondent, and extent of being limited in daily activities because of health. This variable was divided into three ordinal categories: *severe health problems, moderate health problems, and few/no health problems *to examine the dose-response association with the self-reported health status. The independent variables that comprised social structure included *education, economic activity, material deprivation, ethnicity, urban-rural residence*, and *living alone*. These variables were based either on a single item (*education, economic activity, ethnicity, urban-rural residence*, and *living alone*) or on a scale created from responses to several items (*material deprivation*). In the latter case, a summative score was calculated and subsequently divided into categories based on cut-points. The behavioral/attitudinal dimension included *smoking, drinking alcohol, attitude toward healthy lifestyle (*single items), and *low utilization of/access to health care services (*multiple items). The psychological dimension included *depression status, lack of trust in public safety and police, experiencing/witnessing violence*, and *submissive attitude toward men in the household *(all multiple items). The variables in these two dimensions were constructed using a similar approach as in the case of social structure variables. *Depression status *was evaluated through the use of CES-D 20-item self-administered scale [34], the Armenian and Russian versions of which had passed several rounds of forward and backward translations with the final versions pilot-tested.

### Missing data

For single item variables, missing values were treated as missing during the analysis (Additional file 1). After first ensuring the lack of any systematic pattern in the missing data, missing responses to constituent items of multi-item variables were imputed as zero (e.g., lacking that feature). This approach ensured that only a conservative bias (e.g., making it more difficult to detect a significant difference) was introduced. Consistent with the CES-D scale implementation guideline, however, *depression *was treated as missing if any of the 20 component items was missing.

### Statistical methods

The analysis utilized binary logistic regression. The fit of the models was tested using STATA 8.0 statistical package. Most variables were dichotomized or dummy variables were created for ordinal ones to enhance the accuracy of the multivariate analysis. Only age was treated as a continuous variable after examining its linearity on the logistic scale [35]. First, bivariate logistic regression was conducted to identify variables significantly associated with the outcome of poor self-rated health. Next, multivariate logistic regression analyses were performed with variables entered into the analysis in conceptually coherent blocks. Thus, different models were successively used to measure the extent of direct or mediated/controlled effect of each variable on the outcome. Only those variables significantly associated with poor self-rated health during the univariate analysis were retained for subsequent modeling. Model 1 was constructed to measure the "gross effect" of each variable when controlling only for age. Model 2 was constructed to measure the "net effect" when controlling for age and social dimension variables. Model 3 was constructed to measure the "net effect" when controlling for age and the variables included in all three dimensions: social, psychological, and behavioral. And, finally, Model 4 included physical health, a correlate of self-rated health.

To identify the principal determinants of poor self-rated health in women and to detect the possible difference in the sets of determinants between 2001 and 2004, models were fitted separately for the 2001 and 2004 datasets and for the combined dataset. Backward stepwise logistic regression was used, with initial inclusion in the analysis of all those variables associated with the outcome variable at the significance level of = 0.25 (note: *physical health *was excluded from this analysis as it was a correlate of self-rated health and not a potential determinant). Thus, although the association between poor self-rated health and some of the selected variables (*urban-rural residence, lack of trust to public safety/police, experiencing/witnessing violence, drinking alcohol, submissive attitude toward men in the household*, and *attitude toward healthy lifestyle*) was insignificant in the bivariate analysis of the combined data, depending on the level of significance of association between these and the outcome in the different datasets, some of these variables were still included in the stepwise backward logistic regression analysis to fit models in each of 2001 and 2004 datasets and in the combined dataset. The significance level for the Wald statistic was set at 0.05 for initial entry and 0.10 for removal. The final models were assessed by Hosmer-Lemeshow goodness-of-fit test (for 10 groups) and the area under the Receiver Operating Characteristic (ROC) curve [35,36]. The results of the logistic regression analysis are presented as odds ratios (OR) and 95% confidence intervals. For ordinal variables analyzed using dummy variables, the "best" or desired characteristic was used as the referent.

## Results

After controlling only for age (Model 1, Additional file 2), poor self-rated health was significantly associated with the respondents' ethnicity, educational level, economic activity, material deprivation, smoking, low utilization of/access to healthcare services, and depression status. Only ethnicity suggested a protective effect meaning that Armenian women were less likely to rate their health as poor than other ethnic groups. However, this association became marginally significant when controlling for social dimension variables and insignificant when also controlling for behavioral/attitudinal variables and depression (Models 2 and 3, Additional file 2). The relationship between education and self-rated health was significant and remained strong after controlling for age only. A dose-response relationship was also evident: the lower the educational level, the higher the likelihood of rating own health as poor. This association weakened but remained significant after controlling for the social dimension variables, but was extinguished when also controlling for the behavioral/attitudinal and psychological dimension variables (Additional file 2). A similar pattern was observed for economic activity and low utilization of/access to healthcare services. Only age, material deprivation, ever smoking, and depression remained significant in the full model, with dose-response relationships evident for material deprivation and depression.

Physical health and depression measures correlated strongly with self-rated health, with a "dose-response" relationship evident (Additional file 3). When controlling for all the significant variables included in social and behavioral-attitudinal dimensions, the association remained strongly significant (Model 3). When also mutually controlling for depression and physical health (Model 4), the relation between the self-rated health and physical health was altered only minimally, remaining highly significant and preserving the dose-response relationship. Depression behaved similarly, but the strength of the association decreased more than that for physical health, suggesting that mental health indicator was more dependent on the other health correlate than the physical health indicator. These results affirmed the treatment of self-rated health as a comprehensive indicator of health, more informative for physical health status but reflecting also the state of mental health.

### Determinants of poor self-rated health

The final fitted logistic model for the 2001 dataset revealed four significant independent predictors of poor self-rated health: age, education, material deprivation, and depression. The 2004 dataset yielded a slightly different set of determinants: age, material deprivation, depression, and ever smoking. In the combined dataset, the final logistic model included all five variables: age, education, material deprivation, depression, and ever smoking as the main determinants of poor self-rated health. All three models had acceptable calibration and discrimination (Additional file 4). The strength of association between the main determinants and self-rated health differed only slightly in different models. Thus, the models complemented each other with the combined model obtaining enough power to detect all the significant determinants. The combined model showed that only age, material deprivation, and education among social dimension variables were independent predictors of poor self-rated health, with material deprivation showing a strong dose-dependent relationship. Women experiencing severe material deprivation were four times more likely and those experiencing moderate deprivation were twice as likely to rate their health as poor than those not materially deprived. The relationship between educational level and the outcome was moderately strong: those with secondary or less education were 1.7 times more likely to report poor health than those with university or higher education. Ever smoking was the only variable in the behavioral/attitudinal dimension significantly associated with poor self-rated health, with those ever smoking 2.4 times more likely to report poor health than those who never smoked. Among the variables included in psychological dimension, depression status was the only one significantly related to the outcome. Women with probable depression were 2.6 times more likely to report poor health and women with possible depression twice as likely as those with no depression.

## Discussion

This study revealed that health is a resource unequally distributed among women in Armavir *marz*, with those who are materially deprived, are less educated, are depressed, and had ever-smoked more likely to rate their health as poor. While significantly improved between the 2001 and 2004 surveys, the rates of "poor" and the combined "poor" or "fair" perceived health status were problematically high: 80.0% rated their health as "fair" or "poor" in 2001 and 74.6% in 2004, with "poor" cited by 38.1% of women in 2001 and 27.0% in 2004. Women are known to rate their health as poor considerably more often than men [12,16,26,37,38]. Thus, the rates of perceived poor health in this study were compared with that among women in different countries. The rates reported in Armavir well exceeded those in western countries [19,26,39,40], as well countries of Eastern Europe [14,16,41], but were comparable to the rates reported in the Ukraine [12] and Russia [37] during the transition period.

During the study interval, mortality rates stabilized [4], coinciding with significant improvement in socio-economic conditions and a concurrent decrease in the proportion of women rating their health as poor, as the survey data document. Taken together, these findings are consistent with a growing body of evidence suggesting that self-rated health can be predicted by the extent of material deprivation and that socio-economic conditions significantly influence health and mortality patterns [42,43]. In this analysis, a combined variable of material deprivation was used to distinguish among those in different socio-economic strata. This variable included living conditions, possession of convenience items, per-capita expenditures, and the extent of perceived material deprivation, a combination that seemed to differentiate well between varying degrees of material deprivation. The observed strong and independent dose-response relationship between material deprivation and poor self-rated health is consistent with the data from elsewhere [10-12,25,39,44,45].

The strength of the remaining determinants of self-rated health is more modest than that observed for deprivation. The next strongest predictor of perceived health was level of depression. The relation between depression and self-rated health is well established [13,21]. In this study, depression is not only a predictor of poor self-rated health, but also a correlate, related mainly to the psychological aspect of the self-rated health. The slight reduction in the frequency of depression between the two surveys is too modest in comparison with the considerable decrease in the prevalence of poor perceived health status to provide a sufficient explanation for the latter. Instead, the change in depression prevalence itself could be related to improved socio-economic status.

Although the proportion of women who reported ever smoking was small in both samples, the analysis still detected a statistically significant relationship between smoking and poor self-rated health. This finding is consistent with many studies [24,29,46,47]. Carlson [37], however, concluded that smoking did not increase poor self-rated health and mortality rates during the Russian transition and generalized that, in transition periods, changes in perceived health are more closely connected to economic aspects than to unhealthy behaviors like smoking. Given the rarity of smoking among the respondents in this study (6.3% in 2001 and 3.6% in 2004), this behavior cannot be considered as a strong determinant of the substantial reduction of perceived poor health between the two surveys; nor can the data be used to assess Carlson's assertion.

Less education was also a significant determinant of poor self-rated health among women. This finding is consistent with similar studies, including those from post-communistic countries [11,14,16,38,41,45]. The literature suggests several pathways through which lesser education can increase the risk of poor health. Bartley [48] considers that it is not education itself that influences health, but the social position that is obtained through that particular level of education. Wróblewska [16] asserts that those with more education have a greater likelihood of being knowledgeable in terms of health and healthy behaviors. Leinsalu [14] postulates that, in post-communistic countries, the independent effect of education on self-rated health could be better explained by behaviors, problem-solving abilities, values and better coping strategies, because the link between education, occupation and income in these countries is not as consistent as in the West. The present findings support Leinsalu's interpretation as our data shows no correlation between education and material deprivation and the education-related differences in self rated health are smaller than observed in western countries [41,49,50]. Although low educational level is an independent predictor of poor-self rated health, it cannot explain the decline in the prevalence of poor self-rated health between 2001 and 2004, as the level of education was same at both points in time.

This study also produced several unanticipated null findings. First, no urban-rural differences were observed in the perceived health status of women, unlike reports from Ukraine [12] and Estonia [14], where rural residence was associated with poorer self-rated health. Urbanization, however, was not consistently related to health variables in the recent study conducted in Baltic countries and Finland [41]. Second, no relation was observed between a submissive attitude toward men in the household and perceived health. This measure was used as a proxy for women's life control. As the association between life control and health status is well established, particularly in post-communistic countries during the transition period [10-12], this null finding might be explained by the poor fit of this variable as a proxy or, more probably, that in traditional societies, women are more accepting of men making life decisions for them; thus, this is not perceived as being unable to control one's life. Third, no association was observed between perceived health and experiencing/witnessing violence. Previous research indicates that female victims of physical violence are significantly more likely to rate their health as poor [51]. The null finding here might be explained by the lack of differentiation in the survey question between victims of violence and witnesses of it and the small numbers responding affirmatively to these questions. Fourth, the (admittedly weak) proxy for social capital that focused on trust of public officials showed no effect on perceived health status, even though the proportion of those who distrusted public safety and police was substantial in 2001 and increased significantly in the period between the two surveys. Social capital has been demonstrated as crucial for the well-being of a population [29,52]. The proxy variables available in the survey dataset addressed in a limited fashion only formal or "external" aspects of social capital, while important informal constituents such as face-to-face contacts with friends, family members, and neighbors might be more important, especially in a society like Armenia's where individuals are isolated from the state, civil society is lacking, and distrust of the state was solidified during the Soviet period. Similar observations have been made in other former Soviet countries, where informal social ties rather than formal networks were shown to be important for health [10,12]. Fifth, the association between self-rated health and economic activity (including unemployment), living alone, low access to/utilization of health care services, and ethnicity was found to be mediated by other variables and became insignificant in multivariate models.

### Limitations

This study differed from others in terms of how the dependent variable was dichotomized. Unlike the others, only 'poor' ratings were included in poor health, grouping 'less than good' ratings with better health, applying the same approach as Bobak, *et al*. [11], in the study of seven post-communistic countries. There were two reasons for this: the high proportion of respondents rating their health as "poor" and the marked decrease in this proportion between the two surveys, while the decrease in the cumulative proportion of those who rated their health as 'less than good' was not apparent. This finding was judged as evidence of the better sensitivity of the pure 'poor' rating to changes of life circumstances and, thus, made this dichotomization better suited to the aims of the study.

The analysis was based on a combined dataset of two cross-sectional surveys conducted three years apart using independent random samples. The cross-sectional design itself has inherent limitations when attempting to infer causal relationships: misclassification bias may occur when poor socioeconomic conditions result from poor health rather than cause it, and reporting bias may be present, as self-rated health is a subjective measure and thus sensitive to life conditions other than health. Since women in traditional societies like that found in Armavir *marz *usually are not responsible for creating material conditions in their households, the potential selection bias described above is unlikely: their poor health is not inherently linked to adverse socio-economic conditions. Attributing improvement of women's perceived health status to the observed reduction in material deprivation, therefore, seems a plausible explanation. Separately, dissatisfaction with life, rather than poor health itself, might result in rating one's health as poor. This potential reporting bias, however, also can be viewed as a unique feature of the comprehensive nature of self-rated health.

As the combined data are drawn from two different years, response bias may occur due to different response rates and reasons for non-response, as well as historical bias because of possible changes in the set of key determinants of self-rated health since the first survey. Although the response rate was lower in 2004 as compared to 2001, the analysis of the reasons for non-response did not identify any significant differences that might result in biased selection or would make the findings incomparable. Concern about time-dependent changes in the set of key determinants was addressed by analyzing each of the datasets separately, which reassured us that the two datasets yielded comparable findings.

While the samples of surveyed households were representative for Armavir *marz*, women who served as primary respondents were selected to preferably include married women or those having children aged less than 10 years. Thus, older age groups were under-represented in this female population. While older women could present the most vulnerable age group in terms of poor health, depression, and material deprivation, their under-representation in these samples might result in under-estimation of the strength of association between self-reported poor health and its main determinants, suggesting that the true relationship was likely appreciably stronger than that reported here.

Although information on men's health and life was also collected during the surveys, the nature of the dependent variable: self-rated health, made it impossible to include non-primary participants in this study. As a result, we could not make comparisons between genders.

## Conclusion

Overall, the study reaffirms that reduction in material deprivation (a proxy for positive change in socioeconomic conditions) is a strong predictor of improved self-rated health status. Secondarily, changes in the health and social systems and in personal characteristics, such as education, depression, and smoking are also important considerations. Thus, although many challenges to the Armenian health care system remain, decreasing the gap between the rich and poor through social reforms could be a powerful tool for reducing health inequities and improving health status of the population. These lessons should be broadly applicable to CIS countries and others undergoing disruptive economic transitions.

## Competing interests

The authors declare that they have no competing interests.

## Authors' contributions

AD & MET co-conceptualized the original assessment and co-designed the survey instrument. AD oversaw survey administration. AD conceptualized and performed the current analysis and drafted the manuscript. MET contributed to the analysis and critically revised the manuscript. Both authors read and approved the final manuscript.

## Supplementary Material

Additional file 1 Table 1 Distribution (valid percentage) of select social, behavioral/attitudinal, and psychological variables among women aged 18 and over, 2001 & 2004, Armavir *marz*, Armenia Click here for file

Additional file 2 Table 2 The association (p-values and odds ratios (OR) with 95% confidence intervals (CI)) of poor self-rated health with social, behavioral/attitudinal, and psychological dimensions in women aged 18 and over in Armavir *marz*, Armenia, 2001, 2004.* Click here for file

Additional file 3 Table 3 The association (Odds ratios (OR) with 95% confidence intervals (CI)) of physical health and depression with poor self-rated health in women aged 18 and over in Armavir *marz*, Armenia, 2001–2004.* Click here for file

Additional file 4 Table 4 Final models: determinants of self-rated health in women aged 18 and over in the combined dataset and in each of 2001 and 2004 datasets, Armavir *marz*, Armenia Click here for file
