# Supplementary material for: Determinants of self-rated health in women: a population-based study in Armavir Marz, Armenia, 2001 & 2004
Source: Int J Equity Health. 2008 Dec 12;7:25. doi: 10.1186/1475-9276-7-25 (PMC2628913; doi:10.1186/1475-9276-7-25)
Supplement: Additional file 2 — Table 2 The association (p-values and odds ratios (OR) with 95% confidence intervals (CI)) of poor self-rated health with social, behavioral/attitudinal, and psychological dimensions in women aged 18 and over in Armavir marz, Armenia, 2001, 2004.* [file 1475-9276-7-25-S2.doc]

| Table 2. The association (p-values and odds ratios (OR) with 95% confidence intervals (CI)) of poor self-rated health with social, behavioral/attitudinal, and psychological dimensions in women aged 18 and over in Armavir *marz*, Armenia, 2001, 2004.* | | | | | | |
| --- | --- | --- | --- | --- | --- | --- |
|  | Model 1 | | Model 2 | | Model 3 | |
|  | OR | CI | OR | CI | OR | CI |
| Age (continues) | 1.05 | 1.04-1.06 | 1.05 | 1.04-1.06 | 1.05 | 1.04-1.06 |
| Living alone | 1.54 | 0.75-3.18 | 1.10 | 0.51-2.36 | 1.18 | 0.47-3.01 |
| Ethnicity | 0.39 | 0.22-0.69 | 0.56 | 0.31-1.02 | 0.52 | 0.25-1.09 |
| Education |  |  |  |  |  |  |
| School or less | 2.41 | 1.66-3.50 | 1.64 | 1.09-2.46 | 1.52 | 0.95-2.44 |
| Upper secondary | 1.66 | 1.14-2.43 | 1.38 | 0.93-2.06 | 1.32 | 0.83-2.08 |
| University or higher | 1.00 |  | 1.00 |  | 1.00 |  |
| Economic activity |  |  |  |  |  |  |
| Economically inactive | 1.92 | 1.41-2.62 | 1.16 | 0.82-1.64 | 1.36 | 0.89-2.07 |
| Unemployed | 1.97 | 1.46-2.66 | 1.03 | 0.73-1.44 | 1.19 | 0.79-1.80 |
| Employed | 1.00 |  | 1.00 |  | 1.00 |  |
| Material deprivation |  |  |  |  |  |  |
| Severe deprivation | 5.40 | 4.09-7.14 | 4.98 | 3.70-6.69 | 3.84 | 2.64-5.59 |
| Moderate deprivation | 2.12 | 1.66-2.72 | 2.02 | 1.56-2.63 | 1.65 | 1.19-2.28 |
| No deprivation | 1.00 |  | 1.00 |  | 1.00 |  |
| Ever smoking | 2.16 | 1.39-3.35 |  |  | 2.32 | 1.25-4.32 |
| Low utilization of/access to HC | 1.58 | 1.30-1.92 |  |  | 1.17 | 0.90-1.52 |
| Depression |  |  |  |  |  |  |
| Probable depression | 3.78 | 2.72-5.27 |  |  | 2.58 | 1.79-3.71 |
| Possible depression | 2.21 | 1.49-3.27 |  |  | 2.02 | 1.34-3.06 |
| No depression | 1.00 |  |  |  | 1.00 |  |
| *Model 1: Binary logistic regression controlling for age only. Model 2: Binary logistic regression controlling for age, living alone, ethnicity, education, employment, material deprivation. Model 3: Binary logistic regression controlling for age, living alone, ethnicity, education, employment, material deprivation, smoking, access to healthcare services, and depression. | | | | | | |
