# Supplementary material for: Determinants of self-rated health in women: a population-based study in Armavir Marz, Armenia, 2001 & 2004
Source: Int J Equity Health. 2008 Dec 12;7:25. doi: 10.1186/1475-9276-7-25 (PMC2628913; doi:10.1186/1475-9276-7-25)
Supplement: Additional file 3 — Table 3 The association (Odds ratios (OR) with 95% confidence intervals (CI)) of physical health and depression with poor self-rated health in women aged 18 and over in Armavir marz, Armenia, 2001–2004.* [file 1475-9276-7-25-S3.doc]

| Table 3. The association (Odds ratios (OR) with 95% confidence intervals (CI)) of physical health and depression with poor self-rated health in women aged 18 and over in Armavir *marz*, Armenia, 2001-2004.* | | | | | | | | |
| --- | --- | --- | --- | --- | --- | --- | --- | --- |
|  | Model 1 | | Model 2 | | Model 3 | | Model 4 | |
|  | OR | 95% CI | OR | 95% CI | OR | 95% CI | OR | 95% CI |
| Physical health |  |  |  |  |  |  |  |  |
| Severe health problems | 14.72 | 10.55-20.54 | 12.07 | 8.50-17.14 | 12.00 | 8.34-17.25 | 10.44 | 6.89-15.84 |
| Moderate health problems | 3.92 | 2.79-5.49 | 3.62 | 2.55-5.15 | 3.56 | 2.48-5.13 | 3.59 | 2.38-5.42 |
| No health problems | 1.00 |  | 1.00 |  | 1.00 |  | 1.00 |  |
| Depression |  |  |  |  |  |  |  |  |
| Probable depression | 3.78 | 2.72-5.27 | 2.64 | 1.85-3.76 | 2.58 | 1.79-3.71 | 1.89 | 1.28-2.79 |
| Possible depression | 2.21 | 1.49-3.27 | 1.99 | 1.32-2.99 | 2.02 | 1.34-3.06 | 1.43 | 0.92-2.22 |
| No depression | 1.00 |  | 1.00 |  | 1.00 |  | 1.00 |  |
| * Model 1: Controlled for age. Model 2 Controlled for age, ethnicity, living alone, education, employment, material deprivation. Model 3: Controlled for age, ethnicity, living alone, education, employment, material deprivation, smoking, and access to healthcare services. Model 4: Controlled for age, ethnicity, living alone, education, employment, material deprivation, smoking, access to healthcare services, depression, and physical health | | | | | | | | |
