# Supplementary material for: Determinants of self-rated health in women: a population-based study in Armavir Marz, Armenia, 2001 & 2004
Source: Int J Equity Health. 2008 Dec 12;7:25. doi: 10.1186/1475-9276-7-25 (PMC2628913; doi:10.1186/1475-9276-7-25)
Supplement: Additional file 4 — Table 4 Final models: determinants of self-rated health in women aged 18 and over in the combined dataset and in each of 2001 and 2004 datasets, Armavir marz, Armenia [file 1475-9276-7-25-S4.doc]

| Table 4. Final models: determinants of self-rated health in women aged 18 and over in the combined dataset and in each of 2001 and 2004 datasets, Armavir *marz*, Armenia | | | | | | |
| --- | --- | --- | --- | --- | --- | --- |
|  | Combined Dataset  (n =2036, valid n=1415) | | 2001 Dataset  (n=1019, valid n=681) | | 2004 Dataset  (n=1019, valid n=760) | |
| Variable/level | OR | CI | OR | CI | OR | CI |
| Age (years) | 1.05 | 1.04, 1.06 | 1.06 | 1.04, 1.08 | 1.04 | 1.02, 1.05 |
| Education |  |  |  |  |  |  |
| School or less | 1.66 | 1.06, 2.58 | 1.93 | 1.06, 3.54 | - |  |
| Upper secondary | 1.32 | 0.84, 2.06 | 1.43 | 0.78, 2.61 | - |  |
| University or higher | 1.00 | - | 1.00 | - | - |  |
| Material deprivation |  |  |  |  |  |  |
| Severe | 4.19 | 2.93, 5.98 | 3.23 | 1.87, 5.58 | 4.60 | 2.79, 7.58 |
| Moderate | 1.76 | 1.29, 2.39 | 1.62 | 0.98, 2.69 | 1.70 | 1.15, 2.53 |
| No deprivation | 1.00 | - | 1.00 | - | 1.00 |  |
| Depression |  |  |  |  |  |  |
| Probable depression | 2.60 | 1.82, 3.71 | 3.99 | 2.33, 6.83 | 1.83 | 1.14, 2.94 |
| Possible depression | 1.96 | 1.30, 2.96 | 1.94 | 1.05, 3.58 | 2.07 | 1.20, 3.57 |
| No depression | 1.00 | - | 1.00 | - | 1.00 |  |
| Ever smoking | 2.39 | 1.30, 4.42 | - | - | 3.26 | 1.29, 8.24 |
| *Hosmer and Lemeshow goodness of fit test* | *p= 0.321* | | *p= 0.169* | | *p= 0.619* | |
| *Area under ROC curve* | *0.751* | | *0.772* | | *0.726* | |
