# Supplementary material for: Determinants of self-rated health in women: a population-based study in Armavir Marz, Armenia, 2001 & 2004
Source: Int J Equity Health. 2008 Dec 12;7:25. doi: 10.1186/1475-9276-7-25 (PMC2628913; doi:10.1186/1475-9276-7-25)
Supplement: Supplementary file 4 [file 1475-9276-7-25-S1.doc]

Table 1. Distribution (valid percentage) of select social, behavioral/attitudinal, and psychological variables among women aged 18 and over, 2001 & 2004, Armavir *marz*, Armenia

	2001 (%)	2004 (%)	Combined (%)		2001 (%)	2004 (%)	Combined (%)
Urban/rural residence	*n = 1019*	*n = 1019*	*n = 2038*	Low utilization of/access to services**	*N = 1019*	*N = 1019*	*N = 2038*
Urban	39.3	35.3	37.3	Yes	51.1	39.7	45.4
Rural	60.7	64.7	62.7	No	48.9	60.3	54.6
Ethnicity**	*n = 1019*	*n = 1017*	*n = 2036*	Attitude toward healthy lifestyle**	*n = 962*	*n = 959*	*n = 1921*
Armenian	96.3	98.4	97.3	Positive	75.6	80.6	78.1
Other (Russian, Yezidi, other)	3.7	1.6	2.7	Indifferent/negative	24.4	19.4	21.9
Education	*n = 1007*	*n = 1008*	*n = 2015*	Depression*	*n = 700*	*n = 789*	*n = 1489*
School or less	49.5	47.6	48.5	Probable depression	55.4	52.3	53.8
Upper secondary	40.1	42.8	41.4	Possible depression	22.3	19.8	21.0
University or higher	10.4	9.6	10.0	No depression	22.3	27.9	25.3
Economic activity**	*n = 1016*	*n = 1015*	*n = 2031*	Submissive attitude toward men	*N = 1019*	*N = 1019*	*N = 2038*
Economically inactive	32.3	40.6	36.5	Yes	17.0	15.6	16.3
Unemployed	54.2	41.5	47.8	No	83.0	84.4	83.7
Employed	13.5	17.9	15.7	Distrust of public safety/police**	*N = 1019*	*N = 1019*	*N = 2038*
Material deprivation**	*N = 1019*	*N = 1019*	*N = 2038*	Yes	18.6	27.8	23.2
Severe deprivation	29.7	16.0	22.9	No	81.4	72.2	76.8
Moderate deprivation	46.7	39.8	43.3	Experienced/witnessed violence**	*N = 1019*	*N = 1019*	*N = 2038*
No deprivation	23.6	44.2	33.9	Yes	16.9	11.1	14.0
Living alone	*n = 1019*	*n = 1015*	*n = 2034*	No	83.1	88.9	86.0
Yes	1.9	2.0	1.9	Physical health**	*N = 1019*	*N = 1019*	*N = 2038*
No	98.1	98.0	98.1	Severe health problems	39.2	31.0	35.1
Smoking**	*n = 959*	*n = 993*	*n = 1952*	Moderate health problems	37.0	29.7	33.4
Ever smoked/current smoker	6.3	3.6	4.9	No health problems	23.8	39.3	31.6
Never smoked	93.7	96.4	95.1	Self-rated health**	*n = 997*	*n = 979*	*n = 1976*
Drinking alcohol	*n = 906*	*n = 937*	*n = 1843*	Excellent, very good, good	19.9	25.4	22.7
Once a week or more	4.1	3.6	3.9	Fair	41.9	47.6	44.7
Less than once a week	95.9	96.4	96.1	Poor	38.1	27.0	32.6

*Significant difference between baseline and follow-up, p<.05; **significant difference between baseline and follow-up, p<.01; Underlined variables were significantly associated with the outcome (poor self-rated health) during the univariate logistic regression analysis of the combined data; n - number of valid responses; N - total sample (valid for multi-item variables, with missing responses to each constituent item recoded as lacking the feature of interest).
